# Biosorptive Removal of Ethacridine Lactate from Aqueous Solutions by *Saccharomyces pastorianus* Residual Biomass/Calcium Alginate Composite Beads: Fixed-Bed Column Study

**DOI:** 10.3390/ma15134657

**Published:** 2022-07-02

**Authors:** Lăcrămioara Rusu, Cristina-Gabriela Grigoraș, Andrei-Ionuț Simion, Elena-Mirela Suceveanu, Andreea V. Dediu Botezatu, Maria Harja

**Affiliations:** 1 Faculty of Engineering, Vasile Alecsandri University of Bacau, 157 Calea Mărăşeşti, 600115 Bacau, Romania; asimion@ub.ro (A.-I.S.); mirela.suceveanu@ub.ro (E.-M.S.); 2 Faculty of Sciences and Environment, Department of Chemistry Physical and Environment, Dunarea de Jos University of Galati, 111 Domneasca Street, 800201 Galati, Romania; andreea.botezatu@ugal.ro; 3 Faculty of Chemical Engineering an Environmental Protection Cristofor Simionescu, Gheorghe Asachi Technical University from Iasi, 71 A Mangeron Blvd., 700050 Iasi, Romania

**Keywords:** *Saccharomyces pastorianus* residual biomass, immobilization, ethacridine lactate, fixed-bed column biosorption, breakthrough curve, nonlinear regression analysis

## Abstract

In this study, ethacridine lactate removal from aqueous solution using a biosorbent material based on residual microbial biomass and natural polymers in fixed-bed continuous column was investigated. Composite beads of *Saccharomyces pastorianus* residual biomass and calcium alginate were obtained by immobilization technique. The prepared biosorbent was characterized by Fourier transformed infrared spectroscopy, scanning electron microscopy, and analysis of point of zero charge value. Then, laboratory-scale experiments by fixed-bed column biosorption were conducted in continuous system. To this purpose, the column bed high (5 cm; 7.5 cm), initial pollutant concentration (20 mg/L; 40 mg/L), and solution flow through the column (0.6 mL/min; 1.5 mL/min) were considered the main parameters. Recorded breakthrough curves suggest that lower flow rates, greater bed heights, and a lower concentration of ethacridine lactate led to an increased biosorption of the target compound. The biosorption dynamic was investigated by nonlinear regression analysis using the Adams–Bohart, Yoon–Nelson, Clark, and Yan mathematical models. Conclusively, our research highlights, firstly, that the obtained biosorbent material has the required properties for retaining the ethacridine lactate from aqueous solution in continuous system. Secondly, it emphasizes that the modeling approach reveals an acceptable fitting with the experimental data for the Yoon–Nelson, Clark, and Yan models.

## 1. Introduction

Concerns regarding the gradual increase in the concentration of pharmaceuticals in aqueous matrices and their impact on the environment and human health have led to a global research effort to eliminate them from point sources [1,2]. 

The most important sources of pharmaceuticals are wastewater treatment plants, drugs factories, and hospitals, which generate effluents with varying levels of these pollutants [3,4].

As a result, it is critical to remove or separate these compounds; however, existing water and wastewater treatment systems cannot, or can only partially, remove them, due to their resistance to biological degradation [1,5].

Pharmaceuticals that are discharged into the environment can cause toxicity (to varying degrees, depending on the substance in question) at practically any level of the biological hierarchy, including cells, organs, organisms, populations, or ecosystems [6,7].

Despite the fact that the quantities of drugs are much below the medical dosage, there are worries that the presence of pharmaceutical mixes in drinking water may have long-term health repercussions for humans, particularly children and those with impaired immune systems [7].

Various techniques, such as adsorption, biosorption, membrane separation, advanced oxidation processes, electrocoagulation, etc., are increasingly used to eliminate drugs from water [5,8,9,10,11,12,13]. 

Biosorption is the simplest, cheapest, and most adaptable approach for retaining these contaminants among these options [4,14].

Batch and continuous adsorption are two types of adsorption that are based on the operating mode. The first is in a closed system, whereas the second is in an open system with a fixed-bed column [1,15]. 

The equilibrium and kinetic data acquired during batch adsorption are inaccurate for proper design. To acquire realistic information under flow conditions, a continuous operating mode in a fixed-bed column is necessary. The examination of this data is beneficial for determining design parameters and the best operating conditions [16,17].

Adsorption in fixed-bed columns have a number of benefits over batch processes, and may be a feasible choice, since it is a simple mode of operation, can handle large amounts of pollutant solution, and can achieve high treatment efficiency. Moreover, it may be easily scaled-up from a laboratory setting to an industrial application [18,19,20].

From literature surveys, we can conclude that fixed-bed column studies were per-formed for the removal of different types of hazardous pollutants, using various materials as adsorbents (i.e., activated charcoal, lignocellulosic waste material, sludge, biocomposite materials, beach sand, and hydrogel beads) [20,21,22,23,24,25]. In these studies, the breakthrough curves were interpreted to establish the influence of the parameters (e.g., initial adsorption concentration, flow rate, and bed height) on the process, and the experimental data were fitted using the Thomas, Bohart–Adams, Yoon–Nelson, Clark, Yan, and Wolborska models, in order to describe the adsorption process. From the point of view of the number of studies, the results suggest that most research for continuous adsorption processes involve the removal of dyes [25,26,27,28,29] or heavy metals [22,24,30,31,32,33], and there are few references to the removal of pharmaceuticals [34,35,36,37]. 

In this study, a biocomposite material based on microbial biomass and natural polymer was used to remove pharmaceuticals from aqueous solutions in a continuous system. As target molecule was chosen ethacridine lactate, due to the fact that this antimicrobial drug is used in large quantities worldwide. The aim of this study is to investigate the efficiency of a *Saccharomyces pastorianus* residual biomass immobilized in calcium alginate for the removal of ethacridine lactate from water in a fixed-bed column. As far as we know, this paper presents the first examination of the biosorption potential of *Saccharomyces pastorianus*/calcium alginate composite beads for EL removal from aqueous solutions in continuous system.

Different parameters, including the column bed height, initial concentration, and flow rate, were studied for process optimization. Finally, the fixed-bed column adsorption models were analyzed using multiple mathematical models (Bohart–Adams, Yoon–Nelson, Clark, and Yan).

## 2. Materials and Methods

### 2.1. Chemicals and Analytical Procedure 

All reagents were of analytical grade and used as received. 

Ethacridine lactate, potassium dihydrogen phosphate, and disodium hydrogen phosphate were obtained from Merck (Darmstadt, Germany). Hydrochloride acid, sodium chloride, and ethanol were purchased from Chemical Company (Iași, Romania). Sodium hydroxide and calcium chloride were delivered by Chempur (Piekary Ślaskie, Poland). Sodium alginate (low viscosity grade) was bought from BUCHI Laboratortechnik AG (Flawil, Switzerland). 

All solutions were prepared with distilled water. When required, pH was adjusted with NaOH (0.1 M) or HCl (0.1 M).

A 500 mg/L stock solution of ethacridine lactate was prepared and set aside at 4 °C in a closed vessel. Dilutions with concentrations between 1 and 60 mg/L were then obtained; their absorbance, recorded at a wavelength of 431 nm on a UV1280 spectrophotometer (Shimadzu, Tokyo, Japan), served for the construction of a calibration curve.

All the experiments were carried out in triplicate.

### 2.2. Biosorbent Synthesis and Characterization 

Residual biomass of *Saccharomyces pastorianus* was kindly donated by brewing Albrau Company (Onești, Romania). It was kept at −20 °C in sealed plastic bags until use.

The first step of the biosorbent preparation consisted of thawing, washing, decanting, and centrifuging (2500 rpm, 2 × 10 min in a Quirumed 80-2A laboratory centrifuge (Jintan City, China)) this residual biomass. In a second step, a precise quantity of it was introduced in a sodium alginate solution (1% in phosphate buffer of pH 7) for attaining a final concentration of 5%. A thorough homogenization was ensured by magnetic stirring on a Nahita plate (Auxilab, Beriáin, Navarra, Spain). The mixture was dropped in a calcium chloride solution (2%). The resulted beads (SPRBA 5%) were washed with CaCl_2_ 2% and kept in a fresh solution for 24 h at refrigerator (4 °C). The storage solution was removed by washing with distilled water before starting the biosorption experiments.

In terms of biosorbent characterization, scanning electron microscopy analysis (SEM) was the first analysis effectuated. It was conducted on a SEM Quanta 200 3D (FEI Europe B.V., Eindhoven, The Netherlands) apparatus equipped with an energy-dispersive X-ray system. The biosorbent, dried at 50 °C for 2 h in an Air Performance AP60 hot air oven (Froilabo, Paris, France), was positioned to stubs with double adhesive carbon discs. Normal secondary electron mode (SE) in low vacuum was used. A large field detector (LFD) with accelerating voltage of 20 kV, working distance of 14.6–15.5 mm, and spot size of 5 ensured the detection. The magnification range was between 2 mm and 20 μm.

FTIR spectra were collected from 4000 to 400 cm^−1^ (32 sample/background scans; 4 cm^−1^ resolution) with a Nicolet iS50 FTIR spectrometer (Thermo Scientific, Dreiech, Germany) coupled with an ATR accessory. The ATR cleaning was made with ethanol after each spectrum. The reference background spectrum was recorded with air.

For the determination of the point of zero charge, pH_PZC_, sodium chloride was used as background electrolyte. The initial pH (pH_i_) of each NaCl (0.1 M) solution was adjusted between 2 and 12 by small additions of NaOH (0.1 M) or HCl (0.1 M) and measured with a portable pH meter (Dostmann KLH9.1, 0–14 pH, Carl Roth, Karlsruhe, Germany). In each solution (20 mL), 0.4 g of biosorbent was added. After 24 h at room temperature, the final pH (pH_f_) was measured. The pH_PZC_ was established from the curve pH_f_ = f(pH_i_).

### 2.3. Fixed-Bed Column Biosorption 

The adsorption process was conducted in continuous system at room temperature in a glass column (Φ × L = 1.5 cm × 20 cm) with frit on the bottom side. The biosorbent was placed into the column, and EL solution of pH 4 was fed by a Velp Scientifica SP 311/2 peristaltic pump (Monza e Brianza, Italy) in up-flow mode. The effect of different bed depths, EL concentrations, and flow-rates was investigated. EL concentration in the eluted solution was determined by measuring the samples absorbance at the specific wavelength against the calibration curve.

### 2.4. Fixed Bed Column Biosorption Mathematical Modelling

Dynamic performance of the fixed-bed column during the biosorption of ethacridine lactate from aqueous solutions was analyzed with the help of CAVS adsorption evaluation software by applying different mathematical models, such as Adam–Bohart, Yoon–Nelson, Yan, and Clark, to the recovered experimental data. 

## 3. Results and Discussion

### 3.1. Biosorbent Synthesis and Characterization

Alginates are polysaccharides composed of blocks of (1,4)-linked β-D-mannuronic and α-L-guluronic acid residues in different proportions, which influences their physical properties. One of the most exploited of these properties is the ability to form gels by selective binding multivalent cations, such as those of calcium, for example [38]. Based on this consideration, we mixed a sodium alginate solution prepared in phosphate buffer with residual biomass of *Saccharomyces pastorianus* and dropped the suspension in a calcium chloride solution, in order to obtain an adsorptive material employable for the removal of water pharmaceutical contaminants. Spherical, whitish beads resulted from the process. Their mean diameter was 3.218 ± 0.015 mm. 

**Figure 1:**
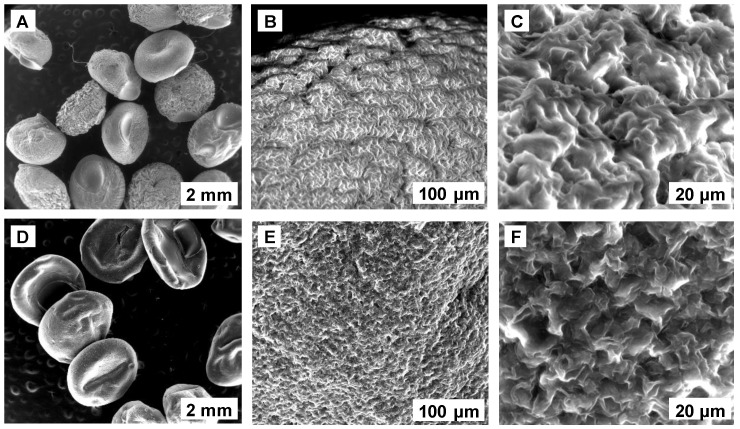
SEM images of SPRBA 5% synthesized biosorbent before (**A**–**C**) and after biosorption (**D**–**F**) of ethacridine lactate from aqueous solution.

SEM micrographs of SPRBA 5% biosorbent are shown in Figure 1. A magnification ratio of 100 μm revealed a uniform surface. When the magnification was fixed to 20 μm, pores with rounded edges were displayed before the biosorption, while, after process completion, a rather sharp shape of the pores was detected, suggesting that the biosorption took place and target compound was retained from the water. 

**Figure 2:**
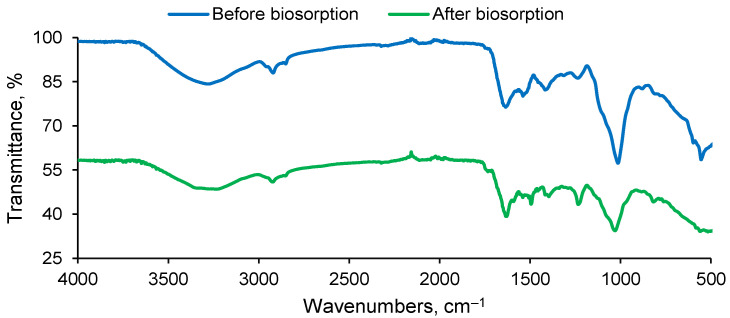
FTIR spectra of SPRBA 5% biosorbent before and after biosorption of ethacridine lactate from aqueous solution.

Along with SEM analysis, FTIR investigation of SPRBA 5% biosorbent was also executed (Figure 2). 

Functional groups of alginate matrix are discernable. Between 3000 and 3200 cm^−1^, vibrations of hydroxyl can be noted. At 2920 cm^−1^, the –CH aliphatic stretching vibration is detected [39]. Asymmetric and symmetric vibrations of carboxyl ions appear from 1600 to 1400 cm^−1^ [40]. C–O (between 1100 and 900 cm^−1^), mannuronate and guluronate residues (1030 cm^−1^), and –CH_2_ bending vibration (1000 cm^−1^) also confirm the polymer structure [41]. Analogous deductions are presented in other papers [42,43,44], in which beads with alginate as polymeric matrix were prepared. Peaks of 1630 and 1540 cm^−1^ can be assigned to amide I and amide II. Between 1300 and 1200 cm^−1^, bands of amide III (proteins) and for PO_2_^−^ (phosphorylated proteins and phospholipids), which possibly caused by the yeast incorporated into the polymeric material, were noticeable [45]. Thus, it can be concluded that residual biomass of *Saccharomyces pastorianus* was incorporated in the resulting adsorbent material. 

The inspection of the spectrum recovered after biosorption reveals an overlapping between the ethacridine lactate specific bands and those of the synthesized biosorbent functional groups. This occurs in the range of 3500 and 3100 cm^−1^ for the N–H asymmetric and symmetric stretching vibrations of aromatic amine and hydrogen-bonded N–H bands, as well as close to 1630 cm^−1^, where the C=N vibrations of the ethacridine lactate acridine ring appears [46]. These findings sustain the fact that the biosorption was favorable and SPRBA 5% prepared was able to remove the ethacridine lactate. 

**Figure 3:**
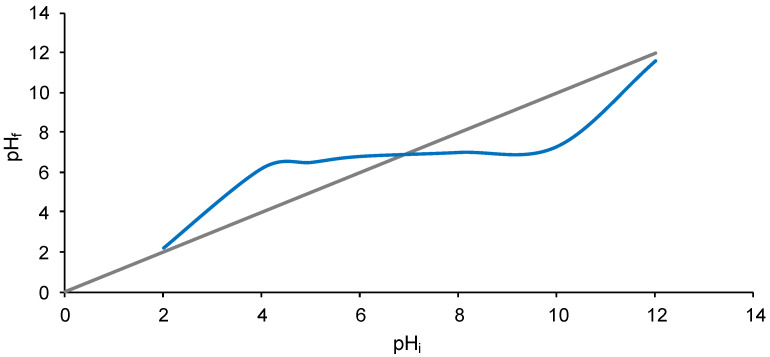
pH_PZC_ of SPRBA 5% biosorbent.

The last analysis applied for the characterization of the biosorbent obtained by the immobilization of *Saccharomyces pastorianus* residual biomass into calcium alginate was represented by the determination of the point of zero charge. pH_PZC_ is conventionally defined as the pH value at which the surface charge is null. The plot pictured in Figure 3 reveals that this point is at pH 6.80. Below this value, SPRBA 5% biosorbent exhibits positive surface charge, while a negative surface charge is encountered above this value.

### 3.2. Impact of Working Parameters on Breackthrough Curves

The amount of EL retained (*q_t_*) by the prepared biosorbent and column adsorption capacity (*q_m_*, mg/g) are given by the following Equations:

(1) $$q_{t}=\frac{Q\cdot C_{0}}{1000}\cdot \overset{t=t_{s}}{\underset{t=0}{\int }}\left(1-\frac{C_{t}}{C_{0}}\right)dt$$

(2) $$q_{m}=\frac{q_{t}}{m}$$

where *Q* is the flow rate (mL/min.), *C*_0_ and *C_t_* are EL initial and at time *t* concentration (mg/L), *t_s_* is the saturation time (min.), and *m* (g) is the mass of the biosorbent packed in the column. 

The mass transfer zone (MTZ, cm) was between 5% and 95% of the inlet concentration variation to effluent concentration and expressed by the Equation (3):

(3) $$MTZ=Z\cdot \frac{\left(t_{s}-t_{b}\right)}{t_{s}}$$

where *Z* is the bed height (cm); *t_b_* and *t_s_* are breakthrough time and saturation time (min.).

The breakthrough volume (*V_b_*, mL), saturation volume (*V_s_*, mL), breakthrough capacity (*q_b_*, mg/g), and saturation capacity (*q_s_*, mg/g) are calculated with Equations (4)–(7).

(4) $$V_{b}=Q\cdot t_{b}$$

(5) $$V_{s}=Q\cdot t_{s}$$

(6) $$q_{b}=\frac{\left(C_{0}-C_{b}\right)\cdot V_{b}}{m}$$

(7) $$q_{s}=\frac{\left(C_{0}-C_{s}\right)\cdot V_{s}}{m}$$

where the *C_b_* (mg/L) is the concentration of effluent at breakthrough point, *C_s_* (mg/L) is the concentration of effluent at saturation point, and the other parameters have the meanings explained above.

The values for the studied parameters (bed height, EL initial concentration, and flow rate) were chosen based on our preliminary studies (data not shown), as well as the previously obtained results in the batch mode [4,5].

**Figure 4:**
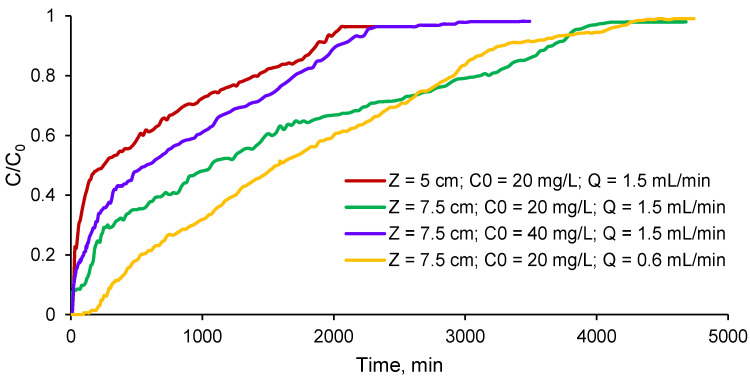
Effect of working parameters on the breakthrough for ethacridine lactate biosorption onto SPRBA 5% biosorbent.

In the first step, two different heights (5 cm and 7.5 cm) for the biosorbent bed were tested, and the results are visible in Figure 4 and Table 1. The pollutant aqueous solution with a concentration of 20 mg/L was passed, with a flow rate of 1.5 mL/min, through the biosorption column. The biosorption capacity augments with the increase of bed height and shows a less steep on the breakthrough curve. For a depth of 5 cm, the bed reached faster the saturation point (after 2035 min). On the contrary, a height of 7.5 cm leads to its increase to 3840 min. The column adsorption capacity was with 57% higher when the bed height was set at 7.5 cm, compared to that registered at a bed depth of 5 cm. 

The second parameter analyzed was the ethacridine lactate initial concentration. Concentrations of 20 and 40 mg/L were selected. The solutions were pumped into the column having the bed depth of 7.5 cm with the same flow rate of 1.5 mL/min. As illustrated in Figure 4, modifications in the breakthrough curve are observable: it is sharper and shifts to origin. With concentration increase, the biosorbent sites are rapidly occupied by the contaminant, with a shorter time period being required to attain the saturation point (2260 min for EL initial concentration of 40 mg/L, compared to 3840 min for EL initial concentration of 20 mg/L). 

The last factor affecting the column biosorption process was the flow rate. Keeping the other parameters at the values established earlier (bed height at 5 cm and EL initial concentration at 20 mg/L), the flow rate was set at 0.6 and 1.5 mL/min. When the higher flow rate is used, the contact time between the EL influent and biosorbent is reduced. Therefore, fewer interactions occur between the contaminant molecule and prepared material. 

A supplementary experiment conducted with a EL solution having an initial concentration at 20 mg/L and flow rate of 0.6 mL/min, but raising the bed depth to 7.5 cm, as exposed in Figure 4, indicates that, at lower EL flow, the interactions between the biosorption bed and target molecule are favorable. The breakthrough point is reached after a longer time (at 100 min), while the saturation point is attained at 4080 min.

Comparable findings, concerning the parameter influences, are reported in other published papers that are dedicated to the removal of various pollutants (including pharmaceutical residues) from aqueous solutions by fixed-bed column adsorption. Kumari et al. [47] studied the process of eliminating fluoride from water with an adsorbent based on sulphuric acid treated alumina and arrived to the conclusion that the fluoride concentration, the bed height and flow rate are important to the defluoridation performance. Aryee and Han [48] investigated the possibility of retaining trimethoprim on a newly prepared biocomposite. Their column adsorption experiments also reveal that parameters such as initial pollutant concentration, bed height, and flow rate strongly influence the adsorption efficiency. Different metals [49,50] were also removed by continuous fixed-bed column adsorption with various adsorbents, and the stated impact of the operating parameters was similar. 

### 3.3. Biosorption Column Modeling

When considering the studied biosorption column parameters, one can note that it is recommended to use the lower flow rate, highest bed depth, and lower ethacridine lactate concentration, in order to attain the best results for dynamic biosorption. In these conditions, the Bohart–Adams, Yoon–Nelson, Clark, and Yan models were used to predict the behavior of ethacridine lactate by fixed-bed column biosorption. 

The Bohart–Adams model describes the relation existing between the *C/C*_0_ ratio and time in the continuous biosorption system of ethacridine lactate on the biosorbent prepared by immobilization of *Saccharomyces pastorianus* residual biomass on calcium alginate. It assumes that the equilibrium is not immediate and, as consequence, the biosorption rate is related to the biosorbent residual capacity and ethacridine lactate concentration [51]. The non-linear form of the Bohart–Adams model is given by Equation (8):

(8) $$\frac{C}{C_{0}}=e^{\left[k_{BA}\cdot C_{0}\cdot \left(t-\frac{N_{0}\cdot Z}{u\cdot C_{0}}\right)\right]}$$

where *C*_0_ (mg/mL) and *C* (mg/mL) are EL concentrations in the influent and in the effluent, *k_BA_* (mL/(mg.min)) is the Bohart–Adams constant, *N*_0_ (mg/mL) is the biosorbent adsorption capacity for unit volume of the bed, *Z* (cm) is bed depth, and *u* (m/min) is the superficial velocity and *t* (min) is the biosorption time. 

According to Hu et al. [52], *k_BA_* affects the flexure of the breakthrough curve, which is more abrupt when this rate constant increases, while an augmentation of *N*_0_ shifts the curve to the right. 

The Yoon–Nelson model is suitable for systems with one molecule to adsorb. It pays no attention to the biosorbent properties and can establish the biosorption capacity and time when the outlet concentration is 50% of the initial one [53]. The Yoon–Nelson model is expressed by the following Equation:

(9) $$\frac{C}{C_{0}}=\frac{e^{\left(t\cdot k_{YN}-\tau \cdot k_{YN}\right)}}{1+e^{\left(t\cdot k_{YN}-\tau \cdot k_{YN}\right)}}$$

where *C*_0_ (mg/mL) and *C* (mg/mL) are EL concentrations in the influent and in the effluent, *k_YN_* (1/min) is the Yoon–Nelson rate constant, *τ* (min) is the time required for 50% breakthrough, and *t* (min) is the biosorption time.

The Clark model associates Freundlich equation and theories of mass transfer [54]. It is given by Equation (10):

(10) $$\frac{C}{C_{0}}={\left(\frac{1}{1+A\cdot e^{r\cdot t}}\right)}^{\frac{1}{n-1}}$$

where *C*_0_ (mg/mL) and *C* (mg/mL) are EL concentrations in the influent and in the effluent, *A* and *r* are Clark parameters, *n* is Freundlich parameter, and *t* (min) is the biosorption time. 

The Yan model is based on the hypothesis that adsorption without coaxial dispersion is directed by a Langmuir pseudo-second-order isotherm [55]. Its form is represented by Equation (11):

(11) $$\frac{C}{C_{0}}=1-\frac{1}{1+{\left(\frac{Q^{2}\cdot t}{k_{Y}\cdot q_{Y}\cdot m}\right)}^{\frac{k_{Y}\cdot C_{0}}{Q}}}$$

where *C*_0_ (mg/mL) and *C* (mg/mL) are EL concentrations in the influent and in the effluent, *Q* is the flow rate (mL/min), *t* (min) is the biosorption time, *k_Y_* (dimensionless) is Yan constant rate, and *q_Y_* (mg/g) is the biosorbent maximum adsorption capacity.

**Figure 5:**
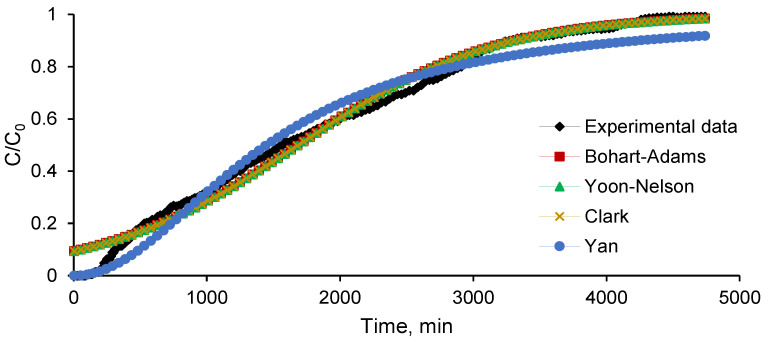
Effect of working parameters on breakthrough curve for ethacridine lactate biosorption onto SPRBA 5% biosorbent.

As summarized in Table 2 and depicted in Figure 5, three of the four applied mathematical models, namely Yoon–Nelson, Clark, and Yan, acceptably fit to the recorded experimental data with a degree of accuracy higher than 0.94. 

Even though there is research [56,57] sustaining that the Bohart–Adams model well-describes only the initial values of the breakthrough curve, our results are not in agreement with this specification (the correlation coefficient was of only 0.608). 

When applied in the process of removing boron by column retention on a modified resin, the Yoon–Nelson model, for example, was able to predict the entire adsorption behavior. Yu et al. [58] simulated the fixed-bed column adsorption of tetracycline on composite materials and provided the idea that the Yoon–Nelson model is suitable for describing the process. On the contrary, Feizi et al. [59], who carried out the adsorption of propranolol, ciprofloxacin, and clomipramide in a fixed-bed column with tyre-based activated carbon, showed that their experimental data were not fitted by this model. Delgado et al. [60] evaluated the applicability of the Yoon–Nelson, Bohart–Adams, Thomas, and modified dose-response models to their experimental records and discovered that they are valuable for obtaining information regarding the fixed-bed column adsorption of carbamazepine and sildenafil on powder-activated carbon. Taking these aspects into consideration, it is worth noting that the studied models can be utilized for the estimation of ethacridine lactate biosorption on the prepared adsorbent material. Similar outcomes were described in the research piloted by Bai et al. [53], who stipulated that the different models are appropriate for different aims. 

## 4. Conclusions

A biosorbent based on *Saccharomyces pastorianus* residual biomass immobilized in calcium alginate was synthesized and characterized. The efficiency of this biosorbent for removing EL from aqueous solutions in fixed-bed column was examined. 

The effects of the column’s functional parameters, such as bed height, EL initial concentration, and flow rate, on the biosorption process were studied. 

The results reveal that, when the bed height was increased from 5 to 7.5 cm, the EL initial concentration was reduced from 40 to 20 mg/L and inlet flow rate was diminished from 1.5 to 0.6 mL/min; the breakthrough point was attained after 300 min, and the time to reach the exhaustion point increased to 4080 min. 

In the continuous system developed in this research work, the biosorption capacity depends on the functional column parameters, a fact that was confirmed by the achieved values, i.e., between 56.234 and 138.584 mg/g. 

The Bohart–Adams, Yoon–Nelson, Clark, and Yan mathematical models were analyzed to predict the breakthrough curves and characterize the EL biosorption process. The relevant parameters of these models were measured by adopting non-linear regression. An acceptable fitting with the experimental data was established for the Yoon–Nelson, Clark, and Yan models. 

Biocomposite beads containing a residual biomass of *Saccharomyces pastorianus* can be an effective biosorbent for ethacridine lactate removal from aqueous matrices. 

In perspective, the benefits of continuous biosorption may be efficiently exploited in scaled-up packed columns for applications in water depollution. 

## Figures and Tables

**Table 1:** Parameters of breakthrough curve for ethacridine lactate removal.

C_0_, mg/L	Z, cm	Q, mL/min	t_b_, min	V_b_, mL	q_b_, mg/g	t_s_, min	V_s_, mL	q_s_, mg/g	q_m_, mg/g	MTZ, cm
20	5	1.5	10	15	1.000	2035	3052.5	74.420	76.116	4.975
20	7.5	1.5	30	45	0.440	3840	5760	118.160	119.546	7.441
40	7.5	1.5	15	22.5	2.120	2260	3390	133.560	138.584	7.450
20	5	0.6	100	60	4.640	3090	1854	66.600	67.124	4.838
20	7.5	0.6	300	180	13.000	4080	2448	55.860	56.234	6.948

**Table 2:** Fixed-bed biosorption column model equations.

Model	*k_AB_*	*N*_0_	*k_YN_*	*τ*	*r*	*A*	*k_Y_*	*q_Y_*	*R* ^2^
Bohart–Adams	0.021554	−1203.15	-	-	-	-	-	-	0.6080
Yoon–Nelson	-	-	0.000975	1296.573	-	-	-	-	0.9623
Clark	-	-	-	-	0.000975	3.54146	-	-	0.9623
Yan	-	-	-	-	-	-	3.569698	1.162059	0.9420

## Data Availability

Not applicable.
